# Hybrid PANI/UiO-66 Thin Film Nanocomposite Membranes with Enhanced Affinity for Heavy-Metal Removal from Drinking Water

**DOI:** 10.3390/membranes16040147

**Published:** 2026-04-14

**Authors:** Zahid Ali, Sana Javed, Tuba Ul Haq, Muhammad Shahid, Noaman Ul Haq, Asim Laeeq Khan

**Affiliations:** 1 Department of Chemistry and Chemical Engineering, Shandong University, Jinan Campus, Jinan 250100, China; 2 Department of Chemistry, The University of Lahore, 1 Kilometer from Defense Road, Lahore 53700, Pakistan; sanaj5055@gmail.com (S.J.); tubaalhaq@gmail.com (T.U.H.); shahid.shahidali404@gmail.com (M.S.); 3 Department of Chemical Engineering, COMSATS University Islamabad, Lahore 54000, Pakistan; noamanulhaq@cuilahore.edu.pk; 4 Department of Chemical Engineering, Faculty of Engineering, Islamic University of Madinah, Madinah 42331, Saudi Arabia

**Keywords:** heavy metals, conductive polymers, forward osmosis (FO), thin film nanocomposite (TFN), metal–organic frameworks (MOF), polyaniline, UiO-66

## Abstract

Heavy metal contamination of drinking water remains a persistent global challenge, exacerbated by salinity, industrial discharge, and the limitations of existing membrane technologies that are constrained by permeability–selectivity trade-offs. In this study, we develop a hybrid thin film nanocomposite (TFN) forward osmosis (FO) membrane by incorporating a zirconium-based metal–organic framework (UiO-66) and its conductive polymer-functionalized analogue (PANI@UiO-66) into the polyamide active layer via interfacial polymerization. The incorporation of UiO-66 enhances water transport through the introduction of hydrophilic microporous domains, while the polyaniline coating modulates nanoscale transport pathways and interfacial interactions. Systematic variation in filler type and loading reveals distinct functional roles of the two fillers. Membranes incorporating bare UiO-66 exhibit increased water flux, attributed to facilitated transport through MOF-derived nanochannels, but show a moderate increase in reverse solute flux. In contrast, PANI@UiO-66 incorporation results in reduced water flux but significantly suppresses reverse solute flux and enhances chromium rejection, indicating improved control over selective transport. At an optimal loading of 0.15 wt% (TFN-PU3), the membrane demonstrates an improved balance between water permeability and solute selectivity compared to the pristine thin film composite (TFC) membrane under FO conditions. The observed performance is attributed to the combined effects of modified transport pathways and interfacial interactions introduced by the hybrid filler system. The results highlight the potential of conductive polymer–MOF hybridization as a strategy for tuning membrane performance. This work provides a practical framework for designing TFN membranes for selective heavy-metal removal in saline and complex water environments.

## 1. Introduction

Global freshwater availability is under accelerating pressure as population growth, urbanization, and rising per-capita water demand converge to exceed the regenerative capacity of natural water systems [1,2,3]. Addressing both freshwater scarcity and water contamination is therefore central to achieving universal access to safe water and sanitation, as articulated in the United Nations Sustainable Development Goals, and demands the development of advanced, energy-efficient, and highly selective water treatment technologies [4]. Environmental monitoring across diverse geographic and industrial contexts consistently identifies toxic heavy metals, most notably chromium (Cr), lead (Pb), arsenic (As), and mercury (Hg), as persistent and high-risk contaminants in drinking water systems [5,6,7,8]. These metals are predominantly released into aquatic environments through industrial activities involving surface treatment, chemical processing, and metallurgical operations, including pigment production, inorganic materials manufacturing, and medical-device fabrication [9]. In particular, Cr is introduced into the environment through both natural processes, such as volcanic activity and weathering of rocks, as well as anthropogenic activities, including industrial discharge, electroplating, leather tanning, and mining operations [10,11]. Cr is highly toxic and can exhibit carcinogenic and mutagenic effects, posing serious risks to human health by damaging the gastrointestinal, respiratory, and nervous systems, along with vital organs such as the liver and kidneys [12,13]. As a result, the effective removal of heavy metals from drinking water has become a critical scientific and technological priority to mitigate environmental and public health risks [14].

A range of physicochemical approaches, such as ion exchange [15], coagulation [16], flotation [17], and photochemical reduction [18], have been deployed for heavy metal removal; however, these methods are often limited by low selectivity, secondary waste generation, high chemical demand, or poor performance under complex water chemistries. Membrane-based separation offers a scalable platform capable of achieving size- and charge-selective transport within a compact process footprint, making it widely applicable across water treatment, gas separation, and emerging hybrid separation systems [19,20]. Forward osmosis (FO) offers a fundamentally different transport route in which water permeation is driven by osmotic pressure gradients rather than externally applied hydraulic pressure, reducing mechanical stress on the membrane active layer [21]. Without externally applied hydraulic pressure, operation mitigates irreversible fouling, enhances membrane longevity, and enables stable solute rejection under high-salinity conditions, positioning FO as a desirable platform for heavy metal removal from saline and industrially impacted waters [22].

Thin film composite (TFC) membranes, comprising a dense polyamide selective layer formed atop a porous support, have become the dominant architecture for high-performance water separation owing to their chemical robustness and favorable permeability–rejection characteristics [23,24]. Despite these advantages, TFC membranes are fundamentally constrained by an intrinsic trade-off between water permeability and solute selectivity, arising from the dense and highly cross-linked nature of the polyamide active layer [25]. To address this limitation, increasing attention has been directed toward thin film nanocomposite (TFN) membranes, in which functional nanoparticles (NPs) are embedded within the active layer to modulate their nanoscale structure, hydrophilicity, and transport pathways [26]. These nanofillers can introduce additional water-conducting channels, suppress fouling through surface energy modification, and enhance selective solute rejection, without compromising membrane integrity [27]. Membrane-based nanocomposite architectures have emerged as highly efficient platforms for selective water treatment [28]. While a wide range of inorganic and carbon-based nanoparticles have been explored as active-layer modifiers, metal–organic frameworks (MOFs) are fundamentally distinct owing to their crystalline porosity, molecularly defined pore architectures, and tunable chemical functionality [29]. UiO-66 is a zirconium-based MOF distinguished by exceptional thermal and chemical stability, arising from strong Zr–carboxylate coordination, and by an intrinsically hydrophilic pore environment [30]. Consistent with this design rationale, Pang et al. demonstrated that incorporating UiO-66 into a graphene oxide–based membrane increased water flux to 29.16 L m^−2^ h^−1^, representing a 270% enhancement over the pristine membrane, highlighting the capacity of UiO-66 to substantially promote water transport when integrated into composite architectures [31].

Although UiO-66 incorporation effectively enhances water permeability, uncontrolled nanochannel formation can increase reverse solute flux, highlighting the need for strategies that restore selectivity without sacrificing transport efficiency. In this context, conductive polymers have emerged as functional modifiers, as their chemical stability and tunable surface charge enable enhanced electrostatic interactions and improved separation behavior in membrane systems [32]. Polyaniline (PANI) is a redox-active conjugated conducting polymer with an amine-rich backbone that provides chemical stability, tunable surface charge through protonation and doping, and strong affinity toward charged species, particularly anionic contaminants [33]. When incorporated into membrane systems, PANI can regulate pore structure, modulate surface wettability, and introduce charge-based selectivity, thereby influencing nanoscale transport behavior and separation performance [34].

Here, we introduce a hybrid TFN FO membrane designed to improve the balance between permeability and selectivity by the deliberate coupling of a highly crystalline UiO-66 with a conductive polymer functional layer. By integrating UiO-66 and its polyaniline-functionalized analogue (PANI@UiO-66) directly into the polyamide active layer via interfacial polymerization, we enable improved control over selectivity within a single membrane architecture. In this design, UiO-66 provides continuous, low-resistance nanochannels that promote water permeation, while the amine-rich, electronically active polyaniline coating regulates pore accessibility, suppresses reverse solute flux, and introduces specific binding interactions for multivalent metal ions. This combined MOF–polymer strategy enables independent tuning of flux and metal rejection, an outcome that is not achievable using either component alone, and establishes conductive polymer–MOF hybridization as a generalizable design principle for selective membrane separations. Using chromium as a model contaminant under saline forward osmosis conditions, we demonstrate that controlled interfacial chemistry, rather than filler loading alone, governs transport selectivity and fouling resistance. Beyond heavy metal removal, this work provides a mechanistically grounded framework for engineering next-generation membranes capable of operating under complex water chemistries where conventional pressure-driven and nanocomposite membranes fail.

## 2. Materials and Methods

### 2.1. Materials

ZrCl_4_ (99.99%), 1,4-benzene di-carboxylic acid (99.99%), aniline hydrochloride (≥98%), ammonium persulfate (≥98%), m-phenylenediamine (MPD) (≥99%), n-hexane (65–70%), hydrochloric acid (HCl) (35%), sodium chloride (NaCl) (99.99%), triethylamine (99.5%), methanol (MeOH) (99.85%), trimesoyl-chloride (TMC) (≥98%), and polyacrylonitrile (PAN) (≥98%) were purchased from Sigma-Aldrich chemicals Pvt Ltd. (St. Louis, MO, USA). N, N-dimethylformamide (DMF) (99.5%) was purchased from Fisher Scientific (Pittsburgh, PA, USA). N-methyl-2-pyrrolidone (NMP) (99.82%) was purchased from Dae-Jung Chemicals (Siheung-si, Republic of Korea). Ammonia solution was purchased from VWR International Pvt Ltd. (Radnor, PA, USA). Alfa Aesar (Haverhill, MA, USA), supplied sodium n-dodecyl sulfate (SDS) (≥99%). All chemicals were of analytical grade and used without any purification.

### 2.2. Synthesis of UiO-66

Initially, 3 g of zirconium tetrachloride and 2.1 g of 1,4-benzenedicarboxylic acid were dissolved in 100 mL of DMF in a round-bottom flask. The mixture was magnetically stirred for 2 h at room temperature to obtain a homogeneous precursor solution. Subsequently, the solution was transferred to a Teflon-lined stainless-steel autoclave and heated in an oven at 120 °C for 12 h under solvothermal conditions to promote the crystallization of UiO-66. After cooling to room temperature, the resulting crystals were collected by centrifugation at 10,000 rpm for 10 min. The obtained solid was washed four times with fresh DMF to remove any unreacted precursors and residual organic ligands, followed by three washes with methanol to exchange the trapped DMF within the pores. Finally, the purified UiO-66 product was dried in an oven at 80 °C for 24 h, yielding approximately 85% of the final material.

### 2.3. Synthesis of PANI@UiO-66

UiO-66 was first activated by drying at 100 °C overnight to remove adsorbed moisture. Separately, an aqueous solution of aniline hydrochloride (5 mM) was prepared and adjusted to pH 2.5 using 0.1 M HCl. An aqueous ammonium persulfate (APS) solution (0.1 M) was prepared as the oxidizing agent. Activated UiO-66 (0.2 g) was dispersed in the acidified aniline hydrochloride solution under gentle mixing to allow adsorption of the monomer onto the MOF surface. Subsequently, 1 mL of the APS solution was added to initiate in situ oxidative polymerization of aniline. The reaction mixture was stirred at room temperature for 12 h to ensure uniform polymer growth on the UiO-66 framework. Following polymerization, the product was treated with aqueous ammonium hydroxide and stirred for 2 h to neutralize residual acidity and stabilize the polymer coating. The resulting PANI@UiO-66 composite was thoroughly washed with deionized water to remove unreacted species and by-products, and then dried at 100 °C overnight. The synthesis procedure follows established literature with minor modifications [35].

### 2.4. Preparation of PAN Support Membranes

Polyacrylonitrile (PAN) support membranes were fabricated via the phase inversion technique. A 16 wt% PAN casting solution was prepared by dissolving PAN (8 g) in NMP under continuous stirring at room temperature for 24 h until a homogeneous solution was obtained. The resulting casting solution was deposited onto a polypropylene/polyethylene (Novavax 2471, Novavax, Gaithersburg, MD, USA) non-woven fabric substrate using a casting knife (Elcometer, Manchester, UK). Immediately after casting, the film was immersed in a deionized water coagulation bath at room temperature to induce phase inversion. After 10 min, the former PAN membrane was removed from the bath and stored in deionized water before further use.

### 2.5. Preparation of TFC and TFN Membranes

TFC and TFN membranes were fabricated via interfacial polymerization on PAN support membranes. The polyamide active layer was formed by reacting m-phenylenediamine (MPD) in the aqueous phase with trimesoyl chloride (TMC) in the organic phase. For TFC membrane preparation, the PAN support was first immersed in an aqueous MPD solution (2 wt%) for 4 min to allow sufficient monomer uptake. Excess solution was removed from the surface, after which the membrane was contacted with an organic solution of TMC (0.2 wt% in n-hexane) to initiate interfacial polymerization.

**Figure 1:**
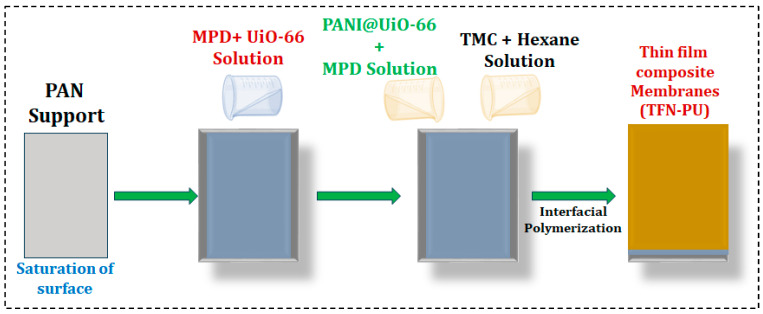
Schematic of the TFN synthesis process.

The reaction was allowed to proceed for 15 min under ambient conditions, forming a dense polyamide selective layer at the aqueous–organic interface. The membrane was subsequently cured at 50 °C for 10 min to stabilize the active layer. TFN membranes were prepared following the same interfacial polymerization protocol, with the exception that UiO-66 or PANI@UiO-66 nanofillers were dispersed in the aqueous MPD solution before membrane formation. Nanofiller loadings of 0.05, 0.10, and 0.15 wt% were investigated for each filler type, yielding three UiO-66–based TFN membranes and three PANI@UiO-66–based TFN membranes (Figure 1). The resulting membranes are denoted according to filler type and loading (Table 1).

## 3. Characterizations

The chemical structures of UiO-66, PANI@UiO-66, and the corresponding TFN membranes were analyzed by Fourier transform infrared (FTIR) spectroscopy over the range 500–4000 cm^−1^ using a Thermo Nicolet 6700 spectrophotometer (Thermo, Madison, WI, USA). Crystallinity and phase purity of MOF were examined by X-ray diffraction (XRD) employing Cu Kα radiation over a 2θ range of 5–80° (PANalytical XPert Powder DY-3805, PANalytical, Almelo, The Netherlands). Surface morphology and cross-sectional structure of UiO-66 and the TFN-PU3 membrane were investigated by field-emission scanning electron microscopy (JEOL FE-SEM (JEOL, Tokyo, Japan), JSM-7800F, 10 kV with secondary detector), accompanied by elemental mapping of UiO-66. The samples were sputter-coated with a thin Au layer to improve conductivity before SEM imaging. The specific surface area and pore-size distribution of UiO-66 were determined by nitrogen adsorption–desorption isotherms measured at 77 K using a Micromeritics ASAP 2460 analyzer (Micromeritics, Norcross, GA, USA).

The separation performance of TFC and TFN membranes was evaluated using a laboratory-scale FO system following established procedures [36]. An aqueous solution of chromium (Cr(VI)) was used as the feed solution, prepared from potassium dichromate (K_2_Cr_2_O_7_), while 1.0 M NaCl served as the draw solution. The membranes were mounted in the FO cell in the active-layer-facing-feed-solution (AL–FS) orientation. All experiments were conducted at room temperature (~25 °C), and each test was performed in triplicate. Chromium rejection was calculated based on mass balance in the feed solution where the concentration was measured by atomic absorption spectroscopy.

Water flux ($J_{v}$) was determined gravimetrically from the change in feed solution mass and calculated according to:

(1) $$\begin{array}{cccc} & J_{v}=\frac{\Delta m/\rho }{\Delta t\times A_{m}} & & \end{array}$$

where Δm is the change in feed solution mass, ρ is the density of the feed solution, Δt is the operation time, and $A_{m}$ is the effective membrane area. Water flux is reported in L m^−2^ h^−1^ (LMH).

Reverse solute flux ($J_{s}$) was quantified by measuring the change in NaCl concentration in the feed solution and calculated as:

(2) $$\begin{array}{cccc} & J_{s}=\frac{C_{f}V_{f}-C_{i}V_{i}}{\Delta t\times A_{m}} & & \end{array}$$

where $C_{i}$ and $C_{f}$ are the initial and final salt concentrations in the feed solution, respectively, $V_{i}$ and $V_{f}$ are the corresponding feed volumes, Δt is the operation time, and $A_{m}$ is the effective membrane area. Salt concentrations were determined using a conductivity meter.

## 4. Results and Discussions

### 4.1. XRD of Filler

**Figure 2:**
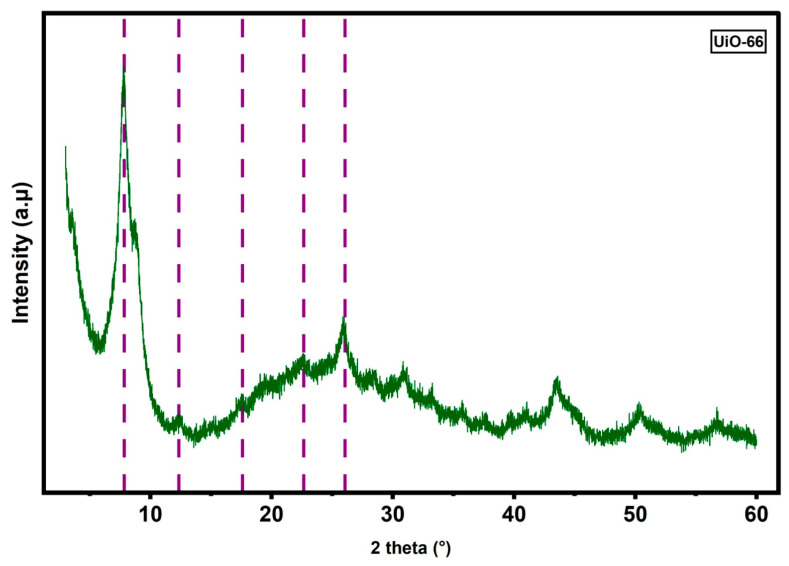
XRD pattern of UiO-66.

Figure 2 presents the XRD pattern of the as-synthesized UiO-66. The diffraction peaks observed at 2θ ≈ 7.5°, 8.6°, 12.3°, and 25.9° are characteristic of the UiO-66 framework and are consistent with previously reported crystallographic patterns [30], confirming the successful formation of the targeted structure. The presence of well-defined reflections at low diffraction angles (7.5° and 8.6°) indicates the preservation of long-range periodicity within the framework, which is a key feature of highly ordered MOF structures. The absence of additional diffraction peaks suggests that no detectable secondary crystalline phases were formed during synthesis. Furthermore, the relatively sharp and distinct nature of the peaks indicates a good degree of crystallinity. Overall, the diffraction pattern confirms the formation of phase-pure UiO-66 with preserved structural integrity under the applied synthesis conditions.

### 4.2. FTIR Analysis of Filler

**Figure 3:**
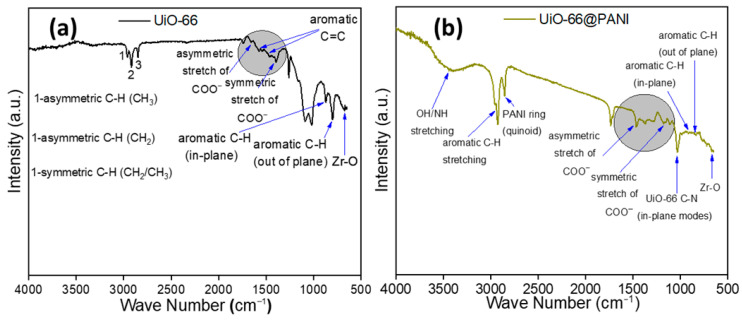
FTIR spectra of (**a**) UiO-66 and (**b**) PANI@UiO-66.

The FTIR spectrum of pristine UiO-66 (Figure 3a) exhibits characteristic vibrational features consistent with a Zr-based carboxylate framework. The absorption band observed at ~746 cm^−1^ is attributed to Zr–O stretching vibrations, confirming coordination between zirconium clusters and carboxylate linkers. The bands at ~1388 cm^−1^ and ~1593 cm^−1^ correspond to the symmetric and asymmetric stretching modes of the carboxylate (–COO^−^) groups, respectively, indicative of deprotonated terephthalate linkers coordinated to Zr nodes. The aromatic skeletal vibrations of the benzene ring appear at ~1505 cm^−1^ and ~1593 cm^−1^. The weak band near ~1660 cm^−1^ is assigned to residual C=O stretching of DMF molecules weakly coordinated within the UiO-66 pores, which is commonly observed in the solvothermal synthesized UiO-66. The absorption bands at ~2850, ~2915, and ~2965 cm^−1^ are associated with symmetric and asymmetric C–H stretching vibrations of aliphatic –CH_2_/–CH_3_ groups, arising from residual solvent or linker-related vibrational modes, and are consistent with reported UiO-66 spectra.

Following polyaniline functionalization, the FTIR spectrum of PANI@UiO-66 (Figure 3b) exhibits distinct additional features characteristic of the conductive polymer coating. The broad absorption band in the range 3200–3400 cm^−1^ is attributed to overlapping N–H stretching vibrations of amine groups in polyaniline and surface hydroxyl groups of UiO-66. The appearance of bands at ~1554 cm^−1^ and ~1480–1500 cm^−1^ corresponds to the quinoid and benzenoid ring stretching modes of polyaniline, respectively, confirming successful in situ oxidative polymerization of aniline on the MOF surface. Notably, attenuation or masking of several UiO-66 framework vibrations in PANI@UiO-66 is observed, particularly in the aliphatic C–H stretching region, which is attributed to the conformal polymer coating rather than structural degradation of the MOF. Importantly, no new bands associated with framework decomposition are detected, indicating that UiO-66 structural integrity is preserved following polymer functionalization.

### 4.3. Morphology and Textural Properties of Fillers

**Figure 4:**
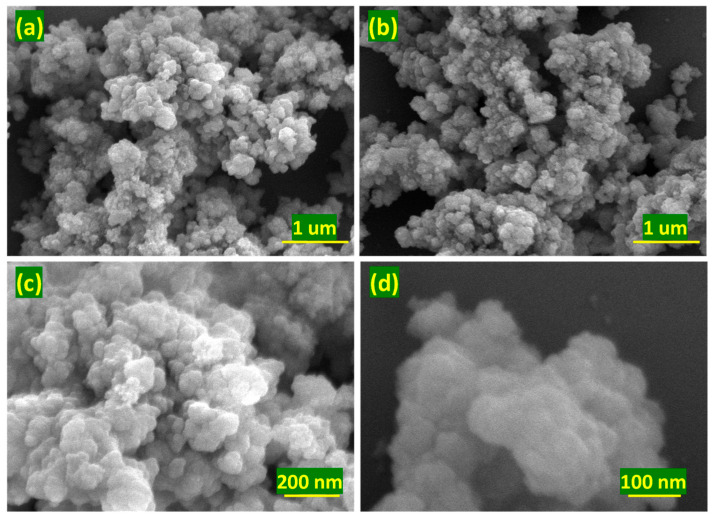
(**a**–**d**) Surface morphology of the UiO-66.

**Figure 5:**
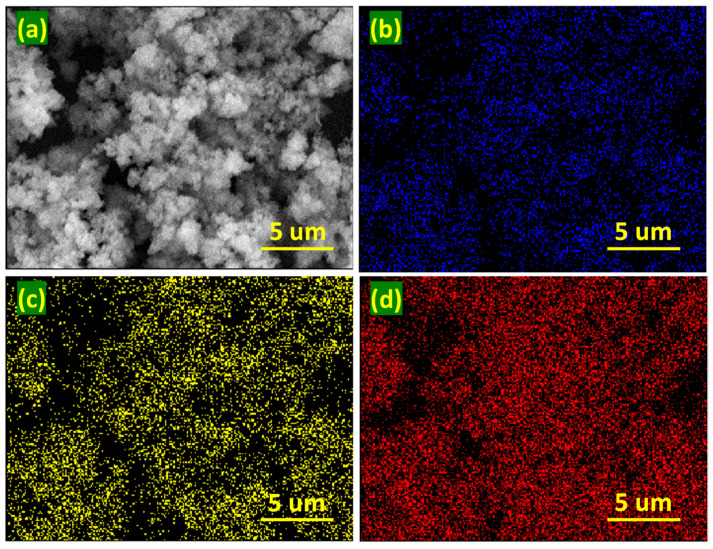
(**a**) Surface morphology of the UiO-66, (**b**) elemental mapping of C, (**c**) elemental mapping of oxygen, and (**d**) elemental mapping of Zr.

The surface morphology and elemental composition of UiO-66 were examined by field-emission scanning electron microscopy (FE-SEM), as shown in Figure 4 and Figure 5. Pristine UiO-66 consists of uniformly distributed, quasi-spherical nanoparticles (Figure 4a–d). The regular particle morphology is consistent with controlled nucleation during solvothermal synthesis and is advantageous for homogeneous dispersion within polymer matrices. Such quasi-spherical nano-crystallites minimize aggregation and reduce stress concentration at the polymer–filler interface, facilitating uniform incorporation into the polyamide active layer during interfacial polymerization. This morphological uniformity is critical for maintaining membrane integrity and avoiding the formation of nonselective voids or interfacial defects. Elemental mapping (Figure 5a–d) confirms the homogeneous distribution of zirconium, oxygen, and carbon across the UiO-66 particles, further verifying the framework’s compositional uniformity.

Nitrogen adsorption–desorption measurements were carried out at 77 K to evaluate the textural properties of the as-synthesized UiO-66. The Brunauer–Emmett–Teller (BET) analysis yields a specific surface area of 833.4 m^2^ g^−1^, which confirms the successful formation of a porous structure with accessible internal surface area. The relatively high surface area suggests that the framework maintains its microporosity after synthesis and post-treatment, which is essential for facilitating mass transport and interaction with permeating species when incorporated into membrane systems.

## 5. Characterization of TFN Membranes

### 5.1. FTIR Analysis of TFN Membranes

**Figure 6:**
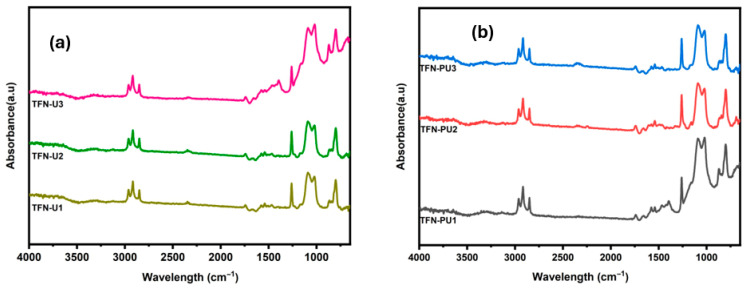
FTIR spectra (**a**) TFN membrane with different loadings of UiO-66 and (**b**) PANI@UiO-66.

FTIR spectra of the TFC and TFN membranes incorporating UiO-66 and PANI@UiO-66 are presented in Figure 6. All membranes exhibit the characteristic amide bands of the polyamide active layer, with amide I (C=O stretching) appearing around ~1660 cm^−1^ and amide II (N–H bending coupled with C–N stretching) observed in the range of ~1500–1540 cm^−1^, confirming the formation of the polyamide selective layer. Following incorporation of UiO-66 or PANI@UiO-66, no prominent new peaks are distinctly resolved in the TFN membrane spectra. This is likely due to the low nanofiller loading and overlap with the dominant polyamide absorption bands. Minor variations are observed in the fingerprint region, but these changes are not sufficiently distinct to allow unambiguous assignment to filler-related vibrations. Similarly, characteristic PANI-related bands are not clearly distinguishable, most likely because of spectral overlap and limited filler content. Overall, the FTIR results mainly confirm the presence of the polyamide active layer, while spectral changes associated with UiO-66 and PANI@UiO-66 incorporation remain subtle.

### 5.2. Morphology of Membranes

**Figure 7:**
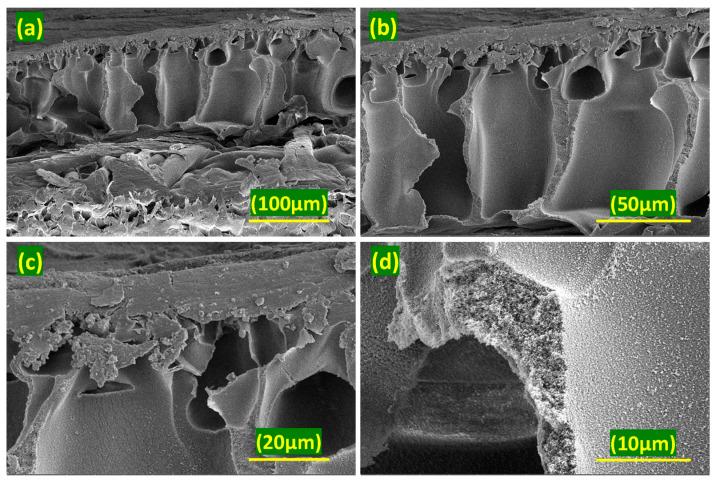
(**a**–**d**) Cross-sectional view of TFN-PU3 membrane.

To examine the morphology of the TFN membranes, cross-sectional SEM analysis was performed on the TFN-PU3 membrane (0.15 wt% PANI@UiO-66), as shown in Figure 7a–d. The membrane exhibits a typical asymmetric structure consisting of a dense polyamide selective layer supported on a porous sublayer with vertically elongated macrovoids. The support layer displays a well-developed porous morphology with interconnected voids and continuous pore walls, which is characteristic of phase inversion–based membranes. At higher magnifications, no obvious macroscopic defects or large agglomerates are observed, indicating that the incorporation of nanofillers does not adversely affect the overall structural integrity of the membrane. The active layer appears continuous and well adhered to the support. Overall, the morphology suggests that the membrane maintains a stable and coherent structure suitable for separation applications.

## 6. Membrane Separation Performance Under Forward Osmosis

### 6.1. Effect of Nanofiller Loading on Water Flux

**Figure 8:**
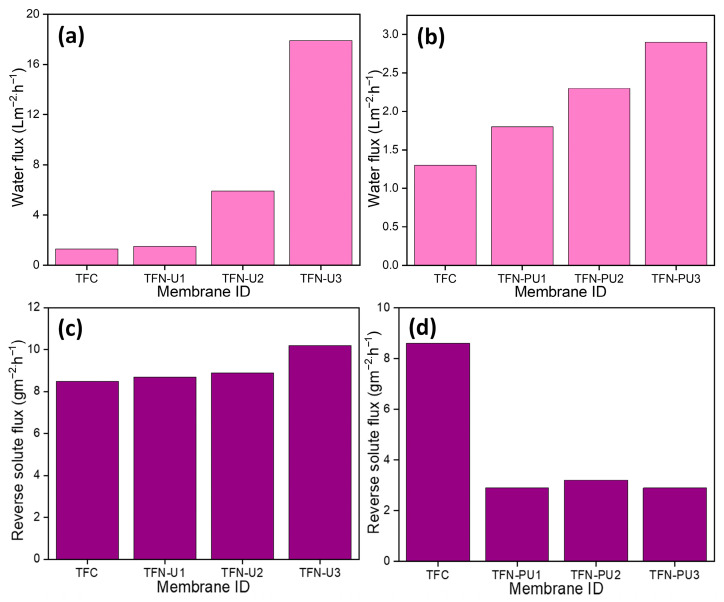
(**a**) Pure water flux of TFC and TFN membranes with different loadings of UiO-66 and (**b**) PANI@UiO-66, reverse solute Flux of TFC and TFN membranes with different loadings of (**c**) UiO-66 and (**d**) PANI@UiO-66.

Water flux is a key indicator of membrane transport efficiency under FO conditions. Figure 8a illustrates the effect of UiO-66 loading on the water flux of TFN membranes. Relative to the pristine TFC membrane, incorporation of UiO-66 leads to a progressive increase in water flux with increasing filler content. At a loading of 0.15 wt% (TFN-U3), the water flux reaches 17.9 L m^−2^ h^−1^, representing a substantial enhancement compared with the unfilled membrane. This increase is attributed to the introduction of hydrophilic, well-defined microporous domains provided by UiO-66, which facilitate water transport across the polyamide active layer. The monotonic increase in flux with UiO-66 loading suggests effective dispersion of the MOF within the membrane matrix and the absence of severe aggregation or transport-limiting defects at the investigated concentrations.

Figure 8b shows the water flux behavior of membranes incorporating PANI@UiO-66. Although water flux increases with increasing filler loading, the overall magnitude remains lower than that observed for membranes containing bare UiO-66, with a maximum value of 2.9 L m^−2^ h^−1^ at 0.15 wt% (TFN-PU3). This comparatively lower flux can be attributed to the presence of the PANI coating, which likely introduces additional resistance to water transport by partially restricting transport pathways. Despite the reduced flux, the incorporation of PANI@UiO-66 is expected to influence membrane selectivity by limiting non-selective transport, highlighting a trade-off between permeability and transport control that is examined further in subsequent sections.

### 6.2. Effect of Nanofiller Loading on Reverse Solute Flux

Reverse solute flux (RSF) is a critical parameter governing the efficiency and selectivity of FO membranes, as it reflects the extent of non-selective ion transport across the active layer. Figure 8c shows the RSF behavior of TFN membranes incorporating bare UiO-66. In contrast to the expectation that additional porous fillers would suppress ion diffusion, a gradual increase in RSF is observed with increasing UiO-66 loading. At 0.15 wt% loading (TFN-U3), the RSF reaches 10 g m^−2^ h^−1^, slightly higher than that of the pristine TFC membrane. This behavior suggests that, while UiO-66 effectively enhances water transport, its incorporation alone does not sufficiently regulate ion diffusion under forward osmosis conditions. The modest increase in RSF is likely associated with the formation of non-selective transport pathways at the filler–polyamide interface, particularly at higher loadings where local aggregation or incomplete interfacial polymerization may occur. Importantly, the increase in RSF remains limited, indicating that UiO-66 does not catastrophically compromise active-layer integrity, but also does not actively suppress reverse salt transport.

A markedly different trend is observed for membranes incorporating PANI@UiO-66 (Figure 8d). Even at low filler loading (0.05 wt%), RSF decreases substantially relative to the TFC membrane, demonstrating effective suppression of solute back-diffusion. A slight increase in RSF is observed at intermediate loading (0.10 wt%), which may arise from local heterogeneities in filler dispersion. However, at the highest loading (0.15 wt%, TFN-PU3), RSF is again minimized, indicating restoration of transport selectivity through improved regulation of the polyamide structure. The superior RSF performance of PANI@UiO-66–modified membranes is attributed to the role of the conductive polymer coating in modulating nanoscale transport pathways. Unlike bare UiO-66, the PANI shell acts as an interfacial regulator during polyamide formation, reducing non-selective free volume and mitigating interfacial defects that serve as ion leakage pathways. This effect results in a denser and more uniform active layer that restricts salt diffusion while maintaining water permeation. Taken together, these results demonstrate that while UiO-66 primarily functions as a permeability enhancer, PANI@UiO-66 enables simultaneous control of water flux and reverse solute flux, effectively decoupling permeability and selectivity in forward osmosis membranes.

### 6.3. Heavy-Metal Rejection Under Forward Osmosis

**Figure 9:**
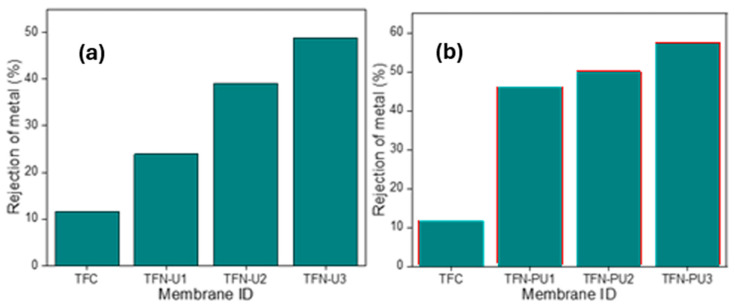
Chromium rejection under different loadings of (**a**) UiO-66 and (**b**) PANI@UiO-66.

The heavy metal rejection performance of the membranes was evaluated under FO conditions. Figure 9a illustrates the chromium rejection behavior of membranes incorporating bare UiO-66. Relative to the pristine TFC membrane, which exhibits limited rejection (~12%), progressive enhancement in chromium rejection is observed with increasing UiO-66 loading. At the highest loading (0.15 wt%, TFN-U3), the rejection reaches approximately 48%. This improvement indicates that incorporation of UiO-66 into the polyamide active layer enhances resistance to chromium transport, consistent with the combined effects of steric hindrance and modified transport pathways introduced by the MOF nanoarchitecture. Notably, the increase in chromium rejection occurs despite a modest rise in reverse solute flux for UiO-66-based membranes, highlighting that rejection is governed by selective transport regulation rather than simple pore blockage. These results reinforce the role of UiO-66 primarily as a permeability-enhancing filler that provides partial selectivity improvement without fully suppressing non-selective ion diffusion.

A slightly pronounced enhancement in chromium rejection is achieved upon incorporation of PANI@UiO-66 (Figure 9b). At identical filler loadings, PANI@UiO-66-modified membranes consistently outperform their UiO-66 counterparts, with the highest rejection (~57%) observed for TFN-PU3. This improvement is attributed to the regulatory effect of the PANI coating, which modifies the nanoscale structure of the polyamide layer and introduces amine-rich functionalities at the membrane–solute interface. This improvement may be associated with interactions between the membrane surface and chromium species, along with modifications in transport pathways introduced by the hybrid filler. Collectively, these results confirm that while UiO-66 improves chromium rejection primarily through permeability-driven pathway modification, PANI@UiO-66 achieves superior separation performance by coupling controlled transport regulation with favorable interfacial chemistry. This distinction highlights the advantage of conductive polymer–MOF hybridization for designing forward osmosis membranes capable of selective heavy-metal removal under saline conditions.

### 6.4. Reusability Test

The operational stability and reusability of the optimized TFN-PU3 membrane were evaluated through repeated FO cycles using chromium-contaminated feed solutions. After each separation cycle, the membrane was retrieved and regenerated by sequential rinsing with deionized water, followed by immersion in 0.1 M HCl for 40 min to remove residual chromium species, and subsequent washing with deionized water until neutral pH was achieved. This regeneration protocol was repeated three times to ensure effective removal of retained metal species. The membrane was then dried at 45 °C for 2 h before reuse.

**Figure 10:**
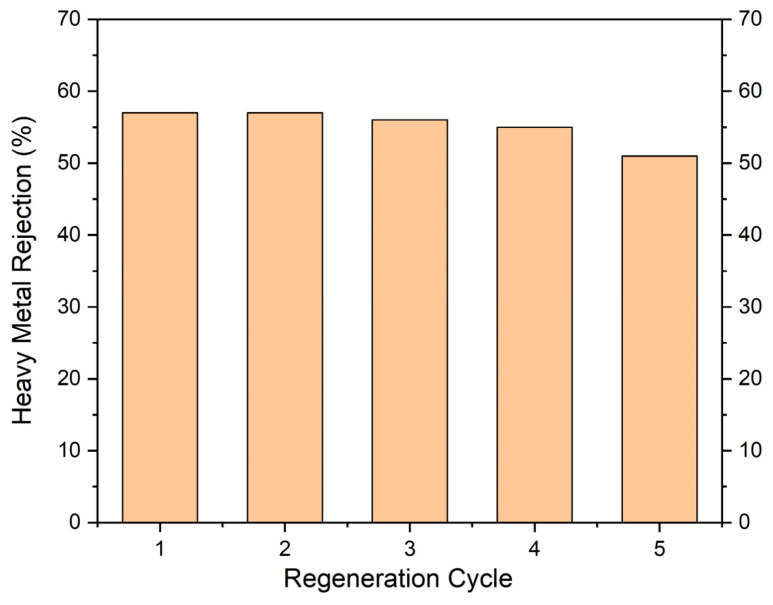
Reusability study of TFN-PU_3_ membrane.

Figure 10 shows the chromium rejection performance of TFN-PU3 over five consecutive FO cycles. The membrane maintains consistently high Cr rejection over the first four cycles, indicating excellent structural and chemical stability under repeated operation and regeneration. A slight decline in rejection is observed in the fifth cycle, which may be attributed to partial fatigue of interfacial functional groups or minor changes in the active-layer microstructure induced by repeated acid exposure. Importantly, no abrupt loss of rejection performance is observed across the tested cycles, demonstrating that the PANI@UiO-66–modified membrane retains its separation functionality under repeated use. These results confirm that the conductive polymer–MOF hybrid architecture not only enhances selectivity but also imparts sufficient robustness for cyclic operation, a critical requirement for practical forward osmosis applications involving heavy-metal-contaminated waters.

### 6.5. Comparison with Literature

Table 2 summarizes the performance of various TFN FO membranes reported for chromium removal under different experimental conditions. Overall, the literature demonstrates that the incorporation of nanomaterials, including MOFs and carbon-based fillers, can significantly influence membrane permeability and selectivity. However, the extent of performance enhancement varies depending on the nature of the filler, membrane structure, and operating conditions. For instance, UiO-66-NH_2_-based membranes have been reported to improve water permeance while maintaining reasonable chromium removal, although the rejection is often presented qualitatively, limiting direct comparison [36]. Similarly, LiCl-modified substrates exhibit very high chromium rejection (≥99.8%), which is primarily attributed to structural densification and reduced solute transport, albeit within a different membrane architecture [37]. Graphene oxide (GO)-based TFN membranes demonstrate a favorable combination of high permeance and chromium rejection, highlighting the role of hydrophilic and layered nanostructures in facilitating water transport while restricting solute passage [38]. MOF-based systems such as MIL-101(Cr) further illustrate the potential of porous fillers to enhance membrane performance; however, the reported chromium rejection (~81%) suggests that achieving high selectivity remains challenging in such systems [5]. More recently, advanced nanofiller designs, such as lignosulfonate-coated ZIF-8, have shown promising results with high water permeance and chromium rejection, reflecting the importance of surface functionalization in regulating transport behavior [39]. In comparison, the present work employs a hybrid UiO-66/PANI system to investigate the interplay between permeability and selectivity in FO membranes. While the observed water permeance is relatively lower than some reported TFN systems, the results highlight the role of controlled filler chemistry in modulating transport pathways and achieving measurable chromium removal under FO conditions. It is important to note that differences in membrane configuration, draw solution, feed composition, and operating parameters across studies limit direct quantitative comparison. Nevertheless, the present approach provides insight into the design of hybrid filler systems for tuning membrane performance, which may be further optimized in future studies.

## 7. Conclusions

In this study, we demonstrate a rational mixed matrix membrane design strategy that decouples water permeability from solute selectivity in forward osmosis through conductive polymer–MOF hybridization. Zirconium-based UiO-66 and its polyaniline-functionalized analogue (PANI@UiO-66) were successfully synthesized, integrated into thin film composite membranes via interfacial polymerization, and systematically evaluated for forward osmosis desalination and heavy-metal removal. Incorporation of bare UiO-66 primarily enhances water transport by introducing hydrophilic, well-defined nanochannels within the polyamide active layer, resulting in a substantial increase in water flux at low filler loadings. However, this permeability enhancement alone provides limited control over reverse solute flux. In contrast, functionalization of UiO-66 with polyaniline introduces an additional level of transport regulation, enabling simultaneous suppression of reverse solute flux and enhancement of chromium rejection under saline forward osmosis conditions. At optimized loading, PANI@UiO-66–modified membranes (TFN-PU3) achieve a balanced performance characterized by improved selectivity, elevated heavy-metal rejection, and stable operation over multiple reuse cycles, highlighting the robustness of the hybrid architecture. These results reveal that conductive polymer functionalization transforms MOFs from permeability enhancers into active regulators of nanoscale transport during polyamide formation. More broadly, this work establishes conductive polymer–MOF hybridization as a generalizable design principle for next-generation mixed matrix membranes, offering a viable pathway to overcome the long-standing permeability–selectivity trade-off in forward osmosis. The insights gained here are directly applicable to the development of advanced membranes for the selective removal of toxic metals from complex saline and industrial wastewaters.

## Figures and Tables

**Table 1:** % Composition of TFC and TFN membranes prepared via interfacial polymerization.

Membrane ID	MPD (%)	TMC (%)	UiO-66 (%)	PANI@UiO-66 (%)
TFC	2	0.2	-	-
TFN-U1	2	0.2	0.05	-
TFN-U2	2	0.2	0.10	-
TFN-U3	2	0.2	0.15	-
TFN-PU1	2	0.2	-	0.05
TFN-PU2	2	0.2	-	0.10
TFN-PU3	2	0.2	-	0.15

**Table 2:** Comparison of FO performance of thin film nanocomposite membranes for chromium removal.

Filler	Polymer	Permeance (L m^−2^ h^−1^)	Chromium Rejection (%)	Ref.
UiO-66-NH_2_	PA (TFC/TFN)	24.8	~High (Cr^3+^ removal, qualitative)	[36]
LiCl	PA (PAN/LiCl substrate)	15.7–30.1	≥99.8 (Cr)	[37]
GO	PA (PSf/PEG support)	34.3	98.3	[38]
MIL-101(Cr)	PA (PAN support)	13	81	[5]
Lignosulfonate-Coated ZIF-8	PA	Improved vs. TFC	98.8 (Cr^3+^)	[39]
PANI@UiO-66	PANI	2.9	57.3	This work

## Data Availability

The original contributions presented in this study are included in the article. Further inquiries can be directed to the corresponding authors.
